# Informing the medical education reform in Tajikistan: evidence on the learning environment at two nursing colleges

**DOI:** 10.1186/s12909-019-1515-0

**Published:** 2019-03-18

**Authors:** Markus Schubiger, Filippo Lechthaler, Mohira Khamidova, Barbara Ann Parfitt, Helen Prytherch, Erik van Twillert, Kaspar Wyss

**Affiliations:** 1 Swiss Tropical and Public Health Institute, Socinstrasse 57, PO Box 4002, Basel, Switzerland; 2 Medical Education Reform Project, Dushanbe, Tajikistan; 3 University of Basel, Basel, Switzerland; 4 Glasgow Caledonian University, Glasgow, UK; 5 School of Agricultural, Forest and Food Sciences, Bern University of Applied Sciences, Zollikofen, Switzerland

**Keywords:** Tajikistan primary healthcare, Medical education, Curriculum reform, Nursing students, Learning environment, Perceptions

## Abstract

**Background:**

The Tajik medical education system is undergoing a complex reform to enhance the transition of the healthcare system from its soviet legacy of emphasizing secondary level care/specialisation to become more family medicine and primary health care oriented. The current study presents the first empirical evaluation of the educational environment for nursing students in Tajikistan using the Dundee Ready Educational Environment Measure (DREEM). The study results contribute to the benchmarking efforts of monitoring and positively steering the educational environment over time.

**Method:**

The study was based on a cross-sectional survey involving 630 nursing students at two nursing colleges in Tajikistan. Students’ perception of the learning environment was measured using the DREEM. Internal consistency was examined using Cronbach’s alpha. General scores were calculated and measured against international benchmarks. Data was further interpreted by comparing DREEM scores between students of different sex, at different colleges and different study years using T tests.

**Results:**

Cronbach’s alpha ranged from 0.30 to 0.75 with an overall alpha of 0.89. General DREEM scores were slightly above average compared to similar studies with nursing students in other countries. In particular, results showed that students’ academic self-perception and teachers’ technical competences were generally favourably rated. Teachers’ pedagogical skills were critically perceived by the study participants and teaching was generally viewed as too teacher-centred with an over-emphasis on factual learning.

**Conclusions:**

Statistical results indicated acceptable levels of reliability of the DREEM tool when applied to the Tajik nursing educational context. Students rated the learning environment as generally satisfactory with average scores similar or slightly higher than comparable scores from similar studies involving nursing students. However, the on-going educational reform could have placed more emphasis on developing faculty pedagogical skills in nursing schools. Teaching approaches would benefit from being more competency based rather than so heavily focused on factual knowledge.

## Background

At present, the medical education system in the Republic of Tajikistan is transitioning from its Soviet legacy - characterized by an overemphasis on factual learning -, to a system which emphasises procedural learning and clinical skills in line with current effective educational practices. The Ministry of Health and Social Protection (MoHSP), as well as the Ministry of Higher Education and their associated institutions have identified medical education reform as a key priority within the current health reform [1]. Various reform plans have already been implemented including a reform of the nursing school curriculum and the establishment of departments of family medicine at the Tajik State Medical University and at several medical/nursing education centres throughout the country [2]. However, medical and health science education is still facing substantial challenges such as lack of sufficient clinical exposure during training, weak pedagogical competencies of faculty, and teaching approaches that emphasise factual knowledge rather than competency based education. Education of nurses in the context of the on-going reforms has not received sufficient attention while the nursing profession continues to be overlooked by the medical society as well as the community as a low-status and low-skilled profession. As a result, many tasks that can be executed by nurses in high-income countries continue to be carried out by physicians in Tajikistan [3]. Moreover, nurses lack a role model and continue to largely be taught by physicians.

In Tajikistan, nurses are trained in nursing colleges – of which the colleges in Dushanbe and Kulob are illustrative. In these colleges the nursing and midwifery tracks follow a common curriculum in the first three years and after that the students enrol in a specialty training for either family nursing or midwifery in the fourth year. Students cannot select the specialty after year three but they have to choose between the midwifery or family nursing faculty when they enter the nursing college.

In high as well as low and middle income countries, innovations in medical and health science education, including the introduction of new pedagogies and their associated faculty development, have become a common practice to enhance the learning environment and boost students’ learning [4–6]. It is generally acknowledged that the learning environment influences students' satisfaction, achievements and success [7–10]. A success that is best demonstrated through improved students’ performance and acquisition of the clinical competences [11]. Measuring changes in the learning environment in the course of an educational reform has hence become a popular tool to monitor the progress and success of medical educations reforms [5]. The most widely used learning environment assessment tool is the Dundee Ready Educational Environment Measure (DREEM), a generic inventory designed to measure the educational environment specifically for medical schools [12, 13]. Different international reviews show that the DREEM tool is a suitable instrument for evaluating the educational environment of medical schools and other healthcare training settings [14, 15]. Despite its relatively frequent use, existing research also reports variable internal consistency of the DREEM’s five scales, and that the construct validity may not be well supported [15]. A systematic review found low validity overall [16], pointing to the critical role of psychometric appraisal when introducing and using the tool.

Building on the experience of two preceding studies conducted with medical students [17, 18], the current paper presents the first comprehensive evaluation of the nursing educational environment in Tajikistan using the DREEM inventory in the context of post-soviet education reform. Although the instrument has already been used for quality assurance in general practice training in Tajikistan [19], to our best knowledge, no consolidated evidence has been presented on its general validity and applicability. In the first step, this paper validated the suitability of the DREEM inventory for the evaluation of nursing education environment in the post-soviet period through testing the internal consistency of the instrument by applying Cronbach’s alpha [15, 20]. In the next step, as a main objective, this study assessed how different aspects of the educational environment in two nursing colleges in Tajikistan (Dushanbe and Kulob) are perceived by family nursing students. Results were stratified and compared between the two colleges, study years (second and fourth year), and sex. The study results will inform current medical education policies in Tajikistan and serve as a reference for monitoring the ongoing educational reforms that foster competency-based education.

## Methods

### Survey design and sampling

The study is based on a cross-sectional survey using the DREEM to evaluate the educational environment at the Dushanbe and Kulob nursing colleges. In addition to the standardized DREEM items, the survey tool includes questions on the participants’ age and sex, as well as an open question at the end, asking students to mention any other issues affecting their educational environment. The questionnaire has been distributed to 327 second-year nursing students and 303 fourth-year family nursing students at Dushanbe and Kulob nursing colleges. The study sample (students) was selected based on the availability of the classes and it has covered all enrolled students in those classes. Paper-based questionnaires were distributed and collected during regular lectures in December 2015 in Kulob and in February 2016 in Dushanbe. The questionnaire process was anonymous and each student response was identified only by a unique number.

### The DREEM tool

The DREEM is a 50-statement closed question questionnaire. It includes 50 items measuring five aspects (sub-scales) of educational environment based on students’ perception: (i) students’ perception of learning, (ii) students’ perception of teachers, (iii) students’ academic self-perception, (iv) students’ perception of atmosphere, and (v) students’ social self-perception. Each item is rated based on five-point Likert-scales capturing students’ degree of agreement with the statement. There are nine negative items that must be scored in a reverse manner prior to data analysis and interpretation. The English version of the DREEM was translated into Tajik using a combined technique as proposed by Cha, Kim and Erlen [21]. The translation required employing three bilingual translators and two rounds of back translation into English for verification. The study tool was pre-tested before use.

### Data analysis

Since this is the first study applying a Tajik version of the DREEM questionnaire and psychometric testing, we tested the internal consistency of the five sub- scales and of the instrument overall using Cronbach’s alpha [20].

Data analysis was widely based on the guidelines by Swift et al. [22] for analysing and reporting of the DREEM. Results were calculated and reported for the aggregated DREEM measure (overall and per year-school group), the five sub-scales (overall and per year group), and individual questionnaire items (overall and per school). Interpretation of the overall score as well as sub-scale scores was done according to McAleer and Roff [23] as shown in Table 1. We compared the sub-scale results between the two schools, across different years within one school, and between male and female study participants using the independent samples T test. P-values < 0.05 were considered statistically significant. As DREEM items typically have bimodal or skewed distributions [24], a central measure like the mean or the median will hide relevant information of a skewed or bimodal distribution such as a high proportion of negative and positive responses. Thus, according to Swift et al [22] the 50 single items were evaluated separately using warnings or so called “flags”. Such items have been discussed separately in addition to the interpretation of the sub-scales. For an item to be flagged, the following thresholds have been considered: (i) The lower threshold for the mean score is 2, indicating areas that need particular attention. The higher threshold is 3, indicating particularly strong areas; (ii) Percentage of answers with “strongly agree/agree” is lower than 50% (higher than 20% for negative items); (iii) Percentage of answers with “disagree/strongly disagree” is higher than 20% (lower than 50% for negative items); and (iv) Percentage of answers “unsure” is higher than 30%. Data analysis was conducted using Excel and the statistical software R. Data was entered into an Excel spreadsheet. Data entry of every fifth questionnaire was double-checked. As quality was found to be sufficient for this subset of data (<1% errors), the remaining 80% of questionnaires were not double checked.

**Table 1:** Interpretation of the overall and sub-scale scores according to McAleer and Roff [23]

Total Score		Perception of learning	
0–50	Very poor	0–12	Very poor
51–100	Plenty of problems	13–24	Teaching is viewed negatively
101–150	More positive than negative	25–36	A more positive perception
151–200	Excellent	37–48	Teaching highly thought of
Perception of teachers		Academic self-perception	
0–11	Abysmal	0–8	Feelings of total failure
12–.22	In need of some retraining	9–16	Many negative aspects
23–33	Moving in the right direction	17–24	Feeling more on the positive side
34–44	Model course organisers	25–32	Confident
Perception of atmosphere		Social self – perception	
0–12	A terrible environment	0–7	Miserable
13–24	There are many issues which need changing	8–14	Not a nice place
25–36	A more positive attitude	15–21	Not too bad
37–48	A good feeling overall	22–28	Very good socially

## Results

### Study population & respondent profile

Six hundred thirty questionnaires were distributed among nursing students at Dushanbe and Kulob nursing colleges. Out of the 630 questionnaires 629 were filled out completely. Table 2 shows the age and sex distribution of the study sample as 73% females with an average age of 20.7 years.

**Table 2:** Study population

Program	Total	Male		Female		Age (SD)
Dushanbe 2nd year	146	27	18%	119	82%	19.7 (1.1)
Dushanbe 4th year	151	41	27%	110	73%	21.8 (1.4)
Kulob 2nd year	181	51	28%	130	72%	19.8 (2.1)
Kulob 4th year	151	52	34%	99	66%	21.7 (2.0)
Total	629	171	27%	458	73%	20.7 (2.0)

### Overall results and results per subscale

Cronbach’s alpha for the total of items was 0.89. Cronbach’s alpha values for students’ perceptions of learning, students’ perceptions of teachers, students’ academic self- perceptions, students’ perceptions of atmosphere and students’ social self-perceptions were 0.68, 0.61, 0.75, 0.72, and 0.30, respectively.

The 50-item DREEM has a score range of 0–200, where 200 marks the ideal educational environment as perceived by the student. The lower most row of Table 3 shows the overall score and the overall score relative to the maximum score for participating students as a whole as well as for those at different colleges and years of study. Total scores for all groups were in the upper half of the maximum score and can thus be interpreted as “more positive than negative” according to McAleer and Roff [23]. Looking at the individual sub-scales, it is important to note that the means for the aggregate sample and that of different student groups did not seem to deviate considerably from each other. In general, students’ academic self-perception was the highest ranked sub-scale, followed by students’ perception of atmosphere, students’ perception of learning, students’ social self-perception and students’ perception of teachers which was the sub-scale with the lowest score on average.

**Table 3:** Mean scores for the five sub-scales of the DREEM instrument for the aggregate sample and per student group

		Overall (n = 629)		Dushanbe				Kulob				Interpretation
				2nd Year (n = 146)		4th year (n = 151)		2nd Year (n = 181)		4th year (n = 151)		
Category / Subcategory	Max Score	Mean (95% CI)	% of max	Mean (95% CI)	% of max	Mean (95% CI)	% of max	Mean (95% CI)	% of max	Mean (95% CI)	% of max	
Students’ perception of learning	48	31.7 (31.2, 32.1)	66.0	32.4 (31.5, 33.3)	67.5	30.1 (29.0, 31.1)	62.6	32.1 (31.3, 32.9)	66.9	32.2 (31.3, 33.1)	67.1	A more positive perception
Students’ perception of teachers	44	27.8 (27.4, 28.2)	63.1	28.1 (27.3, 28.9)	64.0	26.9 (25.9, 27.9)	61.2	28.0 (27.3, 28.7)	63.6	28.1 (27.1, 29.1)	63.8	Moving in the right direction
Students’ academic self-perception	32	23.2 (22.8, 23.5)	72.4	23.5 (22.8, 24.2)	73.5	21.7 (20.9, 22.5)	67.8	23.6 (22.9, 24.3)	73.7	23.8 (23.0, 24.5)	74.3	Feeling more on the positive side
Students’ perception of atmosphere	48	32.4 (31.9, 32.9)	67.6	32.8 (31.8, 33.8)	68.3	31.3 (30.2, 32.3)	65.1	32.9 (32.1, 33.8)	68.6	32.7 (31.6, 33.7)	68.0	A more positive attitude
Students’ social self-perception	28	18.3 (18.1, 18.6)	65.5	18.7 (18.2, 19.2)	66.9	18.2 (17.6, 18.7)	64.8	18.1 (17.6, 18.6)	64.5	18.5 (17.9, 19.1)	66.0	Not too bad
Total Score	200	133.4 (131.8, 135.0)	66.7	135.6 (132.4, 138.8)	67.8	128.1 (124.5, 131.6)	64.0	134.66 (131.9, 137.4)	67.3	135.2 (131.6, 138.8)	67.6	More positive than negative

Table 4 shows the sub scales in more details focusing on items that were flagged as described earlier. For each sub-scale the table shows the mean score of the flagged items, as well as the percentage of respondents who strongly agreed or agreed (SA / A), were undecided (U), or those who disagreed or strongly disagreed (D / SD). A very high percentage of students (95% in Kulob; 88% in Dushanbe) agreed that they are encouraged to participate in class. On the other hand, about half of all students (48%; 54%) found that factual learning was over-emphasized and that their training was too teacher-centred (60%; 54%). Results regarding students’ perception of teachers were somewhat double-edged. On one hand, a high percentage of students considered their teachers to be knowledgeable (87% in Kulob; 84% in Dushanbe). On the other hand, about half of all students stated that teachers get angry in class (46%; 53%) and were irritated by students (48%; 52%). About a quarter of all students stated that teachers did not provide constructive criticism (25%; 24%). Finally, more than 40% of students in both colleges (43%; 41%) disagreed with the statement that teachers are authoritarian. Students’ academic self-perception was generally positive: 86% of the students in Kulob and 85% of students in Dushanbe were confident about their passing, 79% in Kulob and 76% in Dushanbe felt well prepared for their profession, and 87% in Kulob and 80% in Dushanbe agreed that they have learned a lot about empathy in their profession. The response of students’ perception of atmosphere was rather mixed. Most students felt that they were able to ask the questions they wanted (81%, 84%), and the college was perceived as being well timetabled (81% agreed in both colleges). Interestingly, a very high 48% of Kulob students and 41% of Dushanbe students reported finding the overall learning experience disappointing. Furthermore, 29% of Kulob students and 27% of Dushanbe students perceived cheating to be a problem. In response to the question of whether enjoyment outweighed the stress of studying nursing, 25% of Kulob students and 22% of Dushanbe students answered positively. All items measuring students’ social self-perception were flagged. Three items were flagged for having scored higher than 3 on average. In particular, students perceived their friendships at school, their social life, as well as their accommodation as excellent. The other items were primarily flagged because of the bipolar distribution of answers. Thirty percent of Kulob students and 29% of Dushanbe students did not think there was a good support system in place for students suffering from stress. In addition, 21% of Kulob students and 22% of Dushanbe students disagreed with the statement that they rarely got bored in the course. Although the students positively perceived their social life at the college, a considerable percentage of them (35% in Kulob, 25% in Dushanbe) said that they experienced loneliness more often than seldomly. Finally, almost half of the students of both colleges felt too tired to fully enjoy the course.

**Table 4:** Results presented by “flagged items”

#		Kulob				Dushanbe			
	Question	SA/A	U	D/SD	Mean	SA/A	U	D/SD	Mean
Flagged items related to students’ perception of learning									
1 I am encouraged to participate in class		95	2	3	**3.4**	88	5	8	**3.3**
25 The teaching over-emphasises factual learning		**48**	23	**30**	**1.8**	**54**	23	**23**	**1.6**
48 The teaching is too teacher-centred		**60**	20	**20**	**1.8**	**54**	30	**17**	**1.6**
Flagged items related to students’ perception of teachers									
2 The teachers are knowledgeable		87	9	4	**3.2**	84	13	3	**3.2**
8 The teachers ridicule the students		**26**	22	52	2.4	**23**	24	54	2.4
9 The teachers are authoritarian		**36**	21	**43**	2.1	**32**	27	**41**	2.1
32 The teachers provide constructive criticism here		51	24	**25**	2.3	52	**24**	24	2.4
39 The teachers get angry in class		**46**	22	**32**	**1.8**	**53**	22	**26**	**1.7**
50 The students irritate the teachers		**48**	22	**30**	**1.8**	**52**	25	**24**	**1.7**
Flagged items related to students’ academic self-perception									
10 l am confident about my passing this year		86	10	4	**3.2**	85	12	4	**3.1**
21 I feel I am being well prepared for my profession		79	14	8	**3.1**	76	17	7	**3.0**
31 I have learned a lot about empathy in my profession		87	6	7	**3.2**	80	14	5	**3.0**
Flagged items related to students’ perception of atmosphere									
12 This school is well timetabled		81	8	10	**3.2**	81	10	9	**3.0**
17 Cheating is a problem in this school		**29**	25	**46**	2.3	**27**	29	**44**	2.2
35 I find the experience disappointing		**48**	16	**36**	**1.9**	**41**	19	**40**	**1.2**
42 The enjoyment outweighs the stress of studying nursing		54	20	**25**	2.4	51	28	**22**	2.4
49 I feel able to ask the questions l want		81	7	12	**3.0**	84	7	9	**3.1**
Flagged items related to students’ social self-perception									
3 There is a good support system for students who get Stressed		49	21	**30**	2.3	44	29	**27**	2.2
4 I am too tired to enjoy this course		**48**	17	**35**	**1.8**	**48**	19	**33**	**1.8**
14 I am rarely bored on this course		62	17	**21**	2.6	65	13	**22**	2.6
15 I have good friends in this school		91	4	5	**3.3**	91	4	5	**3.4**
19 My social life is good		83	5	12	**3.0**	85	6	9	**3.0**
28 I seldom feel lonely		51	14	**35**	2.2	58	18	**25**	2.5
46 My accommodation is pleasant		84	5	11	**3.0**	83	5	12	**3.0**

Legend: *SA / A* Strongly agree / Agree, *U* Undecided, *D / SD* Disagree / Strongly disagree Values that triggered the flagging are marked in bold numbers

### Comparing student groups

In this section differences in the average scores of the five DREEM sub-scales across student groups are explored. Student responses were compared first across the two different schools, and second across different years within one school. In addition, difference in students’ perception of the educational environment was compared across sex. Table 5 reports the corresponding sub-scale means, p-values and 95% confidence intervals. Despite the small difference in the score magnitude, the scores regarding students’ perception of learning and academic self-perception were statistically significantly higher in Kulob compared to Dushanbe. In Dushanbe, second-year students had a significantly more positive perception of learning, academic self-perception, as well as perception of the atmosphere compared to the fourth- year students. Sub-scale means of Kulob students across different years did not seem to differ significantly. Finally, results showed that there were no significant differences across sex in students’ perceptions captured by the DREEM sub-scales.

**Table 5:** Comparison of sub-scale scores across student groups

	Dushanbe 2nd (n = 146)	Dushanbe 4th (n = 151)	t-test	Kulob _2_nd year	Kulob 4th year (n = 151)	t-test	Male (*n* = 171)	Female (*n* = 458)	t-test	Dushanbe (*n* = 297)	Kulob (*n* = 332)	t-test
Category	Mean score *(SD)*	Mean score *(SD)*	*p*-value (95% CI)	Mean score *(SD)*	Mean score *(SD)*	p-value (95% CI)	Mean score *(SD)*	Mean score *(SD)*	p-value (95% CI)	Mean score *(SD)*	Mean score *(SD)*	p-value (95% CI)
Perception of learning	32.4 (5.4)	30.1 (6.3)	< 0.01 (−3.6, −0.99)	32.1 (5.4)	32.2 (5.8)	0.90 (−1.1, 1.3)	31.9 (5.76)	31.6 (5.8)	0.62 (−1.3, 0.8)	31.2 (5.0)	32.1 (5.6)	0.04 (−1.9, −0.1)
Perception of teacher	28.1 (4.9)	26.9 (6.0)	0.05 (−2.5, 0.02)	28.0 (4.7)	28.1 (6.2)	0.86 (−1.1, 1.3)	27.9 (5.1)	27.7 (5.6)	0.69 (−1.1, 0.7)	27.5 (5.5)	28.0 (5.4)	0.25 (− 1.4, 0.4)
Academic self-perception	23.5 (4.4)	21.7 (4.9)	< 0.01 (−2.9, −0.8)	23.6 (4.9)	23.8 (4.6)	0.69 (−0.8, 1.2)	23.6 (4.9)	23.0 (4.7)	0.13 (−1.5, 0.2)	22.6 (4.7)	23.7 (4.8)	< 0.01 (−1.8, −0.3)
Students’ perception of atmosphere	32.8 (6.2)	31.3 (6.3)	0.03 (−2.9, −0.12)	32.9 (5.8)	32.7 (6.7)	0.69 (−1.7, 1.1)	32.8 (6.6)	32.3 (6.2)	0.39 (−1.6, 0.6)	32.0 (6.3)	32.8 (6.3)	0.11 (−1.8, 0.2)
Students’ social self-perception	18.7 (3.1)	18.2 (3.4)	0.12 (−1.3, 0.2)	18.1 (3.4)	18.5 (3.7)	0.29 (−0.4, 1.2)	18.1 (3.6)	18.4 (3.3)	0.31 (−0.3, 0.9)	18.4 (3.3)	18.3 (3.5)	0.52 (−0.4, 0.7)

## Discussion

As this study presents the first consolidated evidence from a Tajik version of the DREEM questionnaire applied to measure the nursing learning environment, the reliability of the tool has been scrutinized [15]. Generally, it appears that the overall internal consistency of the Tajik version of the DREEM is high, while internal consistency measures of the five sub-scales are somewhat satisfactory, and Cronbach’s alpha value for students’ social self-perception is unsatisfactory. The low internal consistency of the social self-perception sub-scale could be caused by culture-specific variations in response format, as explained by Smith [25]. Further research is needed to explore this issue. In general, there is no consensus over the cut-off level of Cronbach’s alpha for satisfactory scale reliability [26] and it has often been argued that a level of 0.70 is acceptable as described by Nunnally [27]. Others report values higher than 0.50 as being sufficient as in the study by Wang, Zang and Shan [20] that used data from Chinese nursing students and reported alpha values of sub-scales ranging from 0.62 to 0.90, and an overall alpha of 0.95. By applying psychometric testing to the Singaporean version of the DREEM, O’Brien, Chan and Cho [28] reported values ranging from 0.65 to 0.84 for sub-scales (no report of overall consistency).

Results of our DREEM study show that Tajik nursing students at the two nursing colleges perceived their educational environment to be generally satisfactory. With an overall mean score of 133.4, the results of this study were comparable to those (133.5) of DREEM studies among nursing students in Chile [29], China (132.5) [20], and Indonesia (131.0) [10], and slightly higher than studies done in Malaysia (120.1) [30], Egypt (115.0) [31], or Iran (114.3) [32]. The results of the sub-scale measuring students’ academic self-perception scored relatively the highest across all five sub-scales which can be interpreted as students are “feeling more on the positive side” [23]. Three items scored particularly high: first, students felt very well prepared for their profession and, second, they perceived to have learnt a lot about empathy in their profession. Furthermore, students feel confident about passing exams, which might also indicate that exams are not very demanding or that there are flawed examination practices as discussed further below. The average score of the sub-scale on students’ perception of teachers is “moving in the right direction” [23]. Teachers were seen as being quite competent, prepared, and capable of having good interactions with patients. Thus, they were perceived favourably in terms of their technical competence, albeit it should be explained that most of the teachers are physicians rather than nurses themselves, and thus unable to offer a fully apt professional role model to their students. However, this sub-scale has the lowest relative score across the five sub-scales for all student groups. This can be attributed to the fact that students’ perception of teachers’ pedagogical skills was much less favourable and almost half of them stated that the teachers got angry in class and were irritated by students. In addition, about half of students were undecided or disagreed with the statement that teachers provided constructive criticism and about a quarter of them stated that teachers even ridiculed students. These findings highlight the vital need for faculty development programs to improve the teaching skills and ultimately support the learning process. In two items of the “Perception of learning” subscale, students specifically expressed their concerns about the learning process. About half of students thought that teaching over-emphasizes factual learning while more than half of all students perceived teaching to be too teacher-centred. These findings reflect the general teaching approach of the current educational system in Tajikistan. Indeed, most of the teachers themselves have limited nursing experience and are mostly anchored in medical science [33]. The faculty may also lack the skills to teach beyond knowledge recall and hence rather focus on the lower levels of the cognitive domain of Bloom’s taxonomy [34]. In fact, the ongoing reform has mainly focused on changing teaching plans rather than addressing broader aspects of the curriculum such as improving the teaching quality through efficient faculty development programs, and adopting effective pedagogical approaches that support competency based learning. Accordingly, continued efforts are needed to improve the learning process to make the curriculum and the methods of teaching more competency and clinical skills based and to build up a positive nurse role model.

With regard to the atmosphere perceived by students, cheating seems to be an important issue. While the item concerning cheating does not specify what type of cheating is meant, students’ comments at the end of the questionnaire might give an indication to what has driven the low score of this item. Some students commented that examination results were largely affected by students’ outer appearance and particularly by bribing teachers, rather than by students’ performance. This is a serious issue that warrants immediate remedial action to review the examination process and potential faculty involvement in any such flawed practices. Finally, about a quarter of students said that stress levels outweighed enjoyment of the course. Stress can affect memory, concentration, and motivation ultimately leading to decreased learning and academic performance [35]. Thus, it is essential that the learning environment is regularly checked for any potential causes of stress, while students suffering from high levels of stress receive the according support and counselling.

In comparing outcomes of fourth and second year students, this study finds that the average fourth-year family nursing student in Dushanbe generally perceived a more negative environment than the average second-year student. For example, Dushanbe fourth-year students had a significantly lower perception of learning, academic self-perception, and atmosphere when compared to their second- year counterparts. This result is comparable to many DREEM studies from other countries, where students from higher courses poorly perceived the educational environment compared to younger students (see Céron et al. [29] for a review). Some of the authors of those studies suggested that the perception gap is explained by over the years increasing psychological fatigue and the desire of older students to quickly leave their student life behind [29]. A more positive perception on the learning environment was found among students in Kulob as compared to students in Dushanbe for two out of five sub-scores. This result could be linked to differences in the teaching quality between the two nursing colleges. More research is required to identify the drivers of these geographical differences in the perceived learning environment to assure a more equal development of the medical education system in Tajikistan. There was no significant difference comparing the DREEM sub-scale scores across genders.

This study had limitations. As the study tool is not designed to directly observe the learning environment, we relied on self-reporting methods based on the perception of students which bears the risk to over- or understate the actual learning environment. With the DREEM being a standardized tool, we might have not accurately captured the range of factors that affect the learning environment in the Tajik nursing education. Furthermore, existing research shows that validity of the DREEM is not well supported. In order to control this risk, suitability of the DREEM inventory was statistically validated by testing for internal consistency.

## Conclusions

This study presents the first consolidated evidence from a Tajik version of the DREEM questionnaire applied to measure the nursing learning environment. Providing reliable evidence on the learning environment through students’ feedback is vital to ensure: a) a successful implementation of educational policies, and b) an ultimate positive impact on the performance of faculty, students and eventually the graduates. We assessed how different aspects of the educational environment of nursing students in Tajikistan were perceived in order to shed light on strengths and weaknesses of the educational system in place. We showed that the Tajik version of the DREEM revealed statistically acceptable levels of reliability and concluded that the instrument can be considered as an adequate tool for assessing nursing students’ perception of the learning environment. Results indicated that students generally rate the learning environment as favourable with average scores being similar or slightly higher than comparable scores from studies conducted in other countries. Students’ perception on teachers, in particular on their pedagogical skills, revealed to be the most critical aspects of the learning environment. We recommend that the Tajik medical education reform should seriously consider and focus more on faculty development to enhance both the teaching as well as the clinical practice skills. Educational innovations including plans to adopt competency based learning, offering self-directed learning opportunities, and focusing more on clinical skills, are essential steps to reform the medical education system in Tajikistan.
